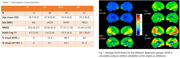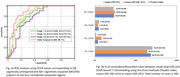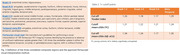# Tau Positivity for Flortaucipir using a Commercial Tau Quantification Software: Comparison to Visual Interpretation

**DOI:** 10.1002/alz.084702

**Published:** 2025-01-09

**Authors:** Rachid Fahmi

**Affiliations:** ^1^ Siemens Medical Solutions USA, Inc., Molecular Imaging, Knoxville, TN USA

## Abstract

**Background:**

18F‐flortaucipir allows in‐vivo visualization of tau tangles in Alzheimer’s disease (AD) with a standardized definition of tau‐PET positivity. However, semi‐quantification is often used in research and in therapeutic trials and plays an important role in the AT(N) framework. This work aims to determine quantitative positivity thresholds and compare semi‐quantification to visual interpretation.

**Method:**

N=189 flortaucipir scans from A05 (NCT02016560) study were included (15 young‐controls (YC), 48 elderly cognitively unimpaired (eCU), 85 MCIs, and 41 with AD) (Table 1), wherein the scans were visually interpreted as either T+/T‐. Using a PET‐only tau quantification software, volume‐weighted SUVRs were calculated on Braak I‐II, Braak III‐IV, Braak V‐VI, and temporal‐meta region (TMR) (Figure 3). Three methods to calculate T+ cut‐points were used: (T1) Youden‐index (MCI/AD vs. Aβ‐ eCU), (T2) mean+2SDs of Aβ‐ eCU, and (T3) mean+2SDs of YC SUVRs. Using calculated cut‐points, a subject is hierarchically assigned to a Braak stage if they are T+ at that stage and at all previous stages, otherwise, the subject is Braak‐staging discordant.

**Result:**

Patients characteristics and visual A+/T+ proportions are summarized in Table 1. T1‐derived thresholds are: 1.26 (Braak I‐II), 1.18 (Braak III‐IV), 1.17 (Braak V‐VI), and 1.29 (TMR) (Table 2). TMR‐based T+ threshold using T2 was 1.30. Overall, the concordance of visual read with TMR‐based quantification was 92%, 93%, and 88.4%, and only 8/189 (4.2%), 6/189 (3.2%), and 0% subjects were Braak staging discordant, using thresholding methods T1, T2, and T3, respectively (Figure 2b). Braak staging was associated with cognitive decline (MMSE and ADAS‐Cog‐11), and with increase in A+/T+ proportions. All Braak V‐VI subjects had a T+ visual read. These subjects were also all Aβ+ MCI/ADs. However, 80% (8/10) of Braak III‐IV, 26.9% (7/26) of Braak I‐II, and 86% (44/51) of TMR+ subjects, using T1‐derived thresholds, had a T+ read, whereas 95.7% (44/46) of TMR+ subjects who were also Aβ+ had a T+ read.

**Conclusion:**

Using the temporal‐meta region, visual read and SUVR were highly concordant. Visual T+ aligns more with amyloid positivity. Hierarchical Braak staging was associated with cognitive performance and amyloid load and its concordance with visual interpretation was moderate‐to‐high for the limbic and neocortical stages.